# Central nervous system tumefactive demyelinating lesions: Risk factors of relapse and follow-up observations

**DOI:** 10.3389/fimmu.2022.1052678

**Published:** 2022-12-01

**Authors:** Xinnan Li, Xiuling Miao, Yaming Wang, Junzhao Sun, Haifeng Gao, Jing Han, Yuxin Li, Qingjun Wang, Chenjing Sun, Jianguo Liu

**Affiliations:** ^1^ Senior Department of Neurology, The First Medical Center of People's Liberation Army (PLA) General Hospital, Beijing, China; ^2^ Department of Neurosurgery, Xuanwu Hospital, Capital Medical University, Beijing, China; ^3^ Senior Department of Neurosurgery, The First Medical Center of People's Liberation Army (PLA) General Hospital, Beijing, China; ^4^ Department of Neurology, Tangshan Gongren Hospital, Tangshan, China; ^5^ Department of Radiology, Sixth Medical Center of People's Liberation Army (PLA) General Hospital, Beijing, China

**Keywords:** tumefactive demyelinating lesions, relapse, etiology, prognostics, multiple sclerosis

## Abstract

**Objective:**

To track the clinical outcomes in patients who initially presented with tumefactive demyelinating lesions (TDLs), we summarized the clinical characteristics of various etiologies, and identified possible relapse risk factors for TDLs.

**Methods:**

Between 2001 and 2021, 116 patients initially presented with TDLs in our hospital were retrospectively evaluated. Patients were followed for relapse and clinical outcomes, and grouped according to various etiologies. Demographic information, clinical data, imaging data, and laboratory results of patients were obtained and analyzed. The risk factors of relapse were analyzed by the Log-Rank test and the Cox proportional hazard model in multivariate analysis.

**Result:**

During a median follow-up period of 72 months, 33 patients were diagnosed with multiple sclerosis (MS), 6 patients with Balo, 6 patients with neuromyelitis optica spectrum disorders (NMOSD), 10 patients with myelin oligodendrocyte glycoprotein antibody-associated demyelination (MOGAD), 1 patient with acute disseminated encephalomyelitis (ADEM), and the remaining 60 patients still have no clear etiology. These individuals with an unknown etiology were categorized independently and placed to the other etiology group. In the other etiology group, 13 patients had recurrent demyelinating phases, while 47 patients did not suffer any more clinical events. Approximately 46.6% of TDLs had relapses which were associated with multiple functional system involvement, first-phase Expanded Disability Status Scale score, lesions morphology, number of lesions, and lesions location (*P<*0.05). And diffuse infiltrative lesions (*P*=0.003, *HR*=6.045, 95%*CI*:1.860-19.652), multiple lesions (*P*=0.001, *HR*=3.262, 95%*CI*:1.654-6.435) and infratentorial involvement (*P*=0.006, *HR*=2.289, 95%*CI*:1.064-3.853) may be independent risk factors for recurrence. Relapse free survival was assessed to be 36 months.

**Conclusions:**

In clinical practice, around 46.6% of TDLs relapsed, with the MS group showing the highest recurrence rate, and lesions location, diffuse infiltrative lesions, and multiple lesions might be independent risk factors for relapse. Nevertheless, despite extensive diagnostic work and long-term follow-up, the etiology of TDLs in some patients was still unclear. And these patients tend to have monophase course and a low rate of relapse.

## Introduction

Tumefactive demyelinating lesions (TDLs) are rare consequences of central nervous system idiopathic inflammatory demyelinating diseases (CNS-IIDD), which can be the initial presentation in various pathological entities with overlapping clinical and radiographic features (1). Most patients with TDLs have or later develop multiple sclerosis (MS) and its variant forms, and a proportion will experience a monophasic course or be diagnosed with neuromyelitis optica spectrum disorders (NMOSD), myelin oligodendrocyte glycoprotein antibody-associated demyelination (MOGAD) or acute disseminated encephalomyelitis (ADEM), and the clinical outcomes of different disease entities are diverse (2–4).

Relapse is a crucial and controversial clinical aspect of demyelinating diseases, and it not only accelerates the course of the patient’s condition but also places a significant psychological load on them (5). Identifying and managing relapse risk factors as well as early diagnosis of TDLs with a variety of etiologies are thus critical issues that must be addressed. However, data on the long-term prognosis of individuals with TDLs are limited. Some researchers believe that TDLs are mostly single-phase disease course, with good prognosis and rare recurrence (6). Others believe that TDLs is a subtype of multiple sclerosis, with a high recurrence rate (7). Jeong et al. followed up 31 cases of TDLs for at least one year (median follow-up time 37.6 months), and 18 cases (58.1%) experienced a second attacks (8). Altintas and colleagues found that 16.7% of TDLs had relapses within 38 months of follow-up, which could recur as TDLs, MS, or NMOSD. However previous studies have mostly been done in small series with short follow-up periods (9). As for the long-term outcome is concerned, available data are insufficient to draw any conclusion.

Therefore, the primary purpose of our study was to assess the long-term outcomes of TDLs with varied etiologies by clinical follow-up and to identify possible relapse risk factors.

## Methods

### Clinical data collection

The study collected and analyzed the clinical follow-up data of patients with TDLs diagnosed in the Sixth Medical Center of PLA General Hospital from January 2001 to December 2021. Inclusion criteria were (1): pathological confirmation or clinical confirmation according to the Chinese TDLs diagnostic criteria (10). (2) With TDLs as the first onset diagnosis. (3) Authorization for informed consent was obtained from the patients or their families. (4) All cases were followed up for ≥ 6 months. Patients who experienced inflammatory demyelinating phases of the CNS previous to presenting with TDLs were specifically excluded. In addition, patients of tumor, infection, vascular or other non-demyelinating inflammatory CNS diseases were eliminated, as well as a history of brain irradiation. Ultimately, 116 patients were enrolled due to inclusion criteria. The collected data included the following contents: (1) Demographic and clinical data: age, gender, first onset disease course, clinical syndrome, Expanded Disability Status Scale (EDSS) at presentation, medical history, poor appetite, and biopsy or not. (2) Laboratory data: lumbar puncture pressure, Cerebrospinal fluid (CSF) routine, CSF biochemical, CSF cytological, serum and CSF oligoclonal band (OCB), serum anti-aquaporin 4 (AQP-4), serum anti-myelin oligodendrocyte glycoprotein (MOG). (3) Imaging data: lesions morphology (i.e. infiltrative, ring-like, megacystic, Balo-like) (11), number of lesions, intracranial maximum lesions diameter on T2WI, localization (i.e. cortex/subcortical, periventricular white matter, deep gray matter, or infratentorial), and data on spinal cord MRIs, when available.

### Pathological material

Histopathology was available in 72 cases. All biopsies were conducted between January 2001 and December 2021 and at least one board-certified neuropathologist assessed the material in each case. In most cases, it was impossible to pinpoint the precise indication for a brain biopsy because the majority of biopsy specimens were obtained retroactively, several years after the date of the biopsy, and clinical follow-up was not carried out by the clinician who was initially involved in the patient’s consultation at the time of the biopsy.

### Aetiological classification

Two neurologists with more than 12 years of experience categorized the TDLs patients jointly and reached a consensus to divide into six etiology-based categories: MS, NMOSD, MOGAD, Balo, ADEM, and the other etiology (8). Patients who had previously been diagnosed with Balo but at the time of a relapse met the diagnostic criteria for MS were classified as MS group.

### Follow-up forms and Relapse

Patients were followed up by outpatient, telephone, or hospitalization. Recurrence was the end point of follow-up. Relapse free survival (RFS) was the time from onset to recurrence, and the time of death from other diseases or no recurrence at the end of follow-up was the end value.

### Statistical methods

The measurement data that conformed to normal distribution were expressed as mean ± standard deviation (X ± S), whereas those that skewed distribution were expressed as median (quartile). And the counting data was expressed by percentage. The association between each variable and the outcome was evaluated using the Kaplan Meier method and the log rank test for survival analysis. The statistically significant log rank test factors were then used as variables in the Cox proportional hazards model for multivariate analysis to figure out the final influencing factors. We used a *p* < 0.05 as the threshold of statistical significance.

## Results

### Clinical data

The average age at presentation was 37.3 ± 14.1 years (range 5–73 years), and there was no significant difference in gender (52.6% female and 47.4% male). The median course of disease and median EDSS score at presentation were 2 months (range 1–72 months) and 3 points, respectively. Fewer patients were current or previous smokers (13.8%) or alcoholics (7.8%).

Initial symptoms and clinical symptoms of patients were summarized, with headache (27.6%, 13.4%), limb numbness (21.6%, 16.1%) and hemiplegia (19.0%, 20.5%) being most common, followed by dysarthria (8.6%, 10.2%), visual impairment (7.8%, 11.8%), epilepsy (4.3%, 2.7%), mental symptoms (4.3%, 6.3%), cognitive impairment (3.4%, 5.1%), aphasia (1.7%, 0.8%), dizziness (0.9%, 9.5%), and dysuria (0.9%, 3.9%). According to the EDSS score, the function of the nervous system was classified as pyramidal, cerebellar, brainstem, sensory, bowel & bladder, visual, cerebral, and others, and 80 patients (69.0%) had multisystem functional impairment in our study.

### Laboratory findings

The median lumbar puncture pressure of cerebrospinal fluid (CSF) in 70 patients was 165mmH_2_O, which rose in 23 participants; the median white blood cell (WBC) count in CSF was 2×10^6^/L, which increased in 13 cases; and the median CSF protein concentration was 368mg/L, which increased in 23 cases. The median glucose level was 3.5 mmol/L, and 9 patients were abnormal; the median chloride level was (122.2 ± 4.5) mmol/L, and 23 cases were abnormal. 19 out of 70 patients were OCB positive. On the basis of clinical suspicion of NMOSD, AQP4 antibody serology was done on 78 participants and 5 of them were positive. 10 of the 30 participants who underwent MOG antibody serology obtained a positive result and were subsequently diagnosed with MOGAD.

### Pathological findings

**Figure 1:**
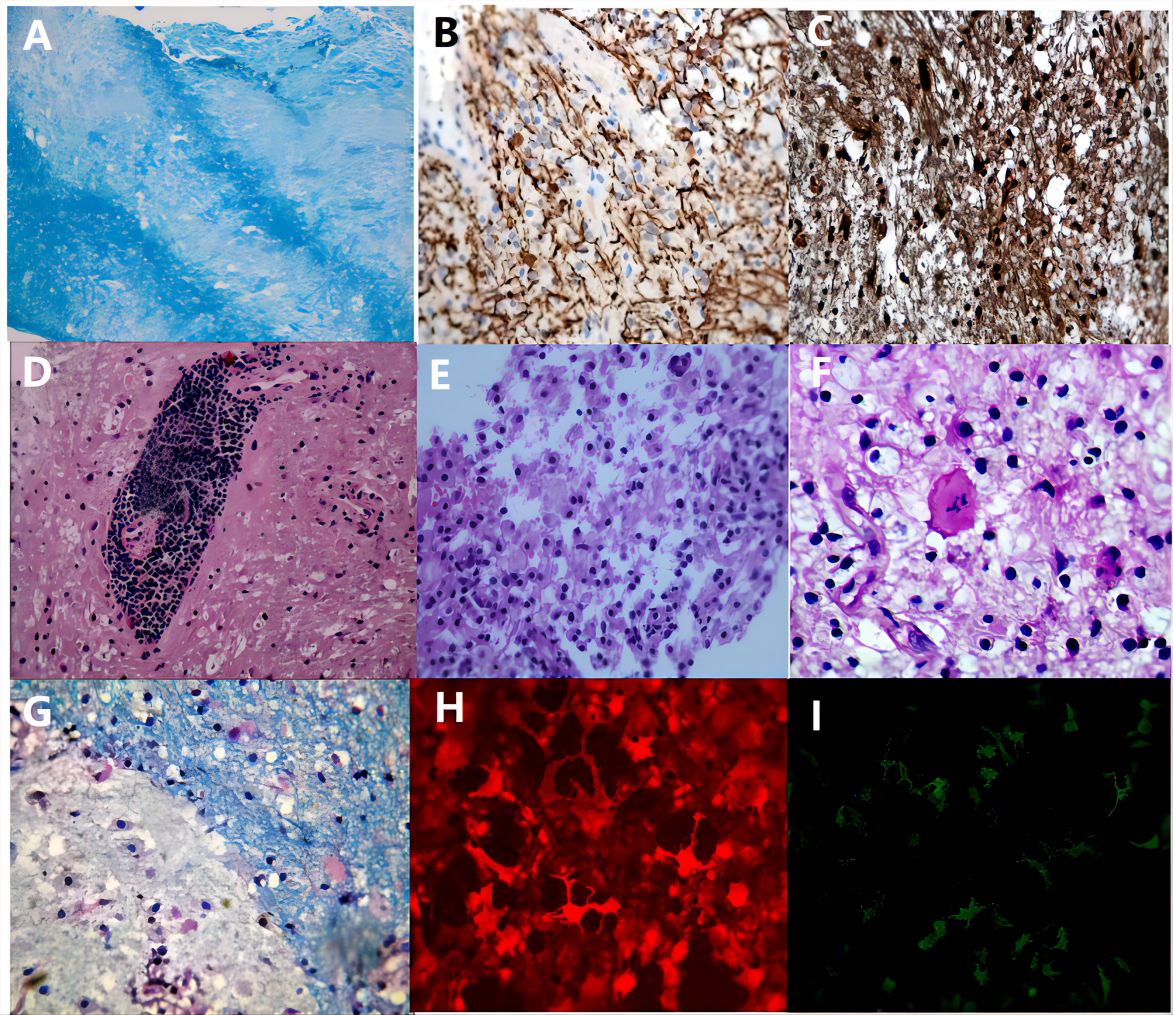
The pathological manifestations of TDLs. The inflammatory demyelinating lesions in 72 patients had the following characteristic manifestations: All patients had myelin loss, a laminar structure between myelin loss and myelin preserved area was seen in Balo patients **(A)**, luxol-fast blue, x200); a few patients with severe CNS inflammation had a reduced number of axons and relatively preserved axons **(B)**, neurofilament, x200); most patients had preserved but swollen axons **(C)**, neurofilament, x200); lymphocyte “sleeve” around the blood vessels **(D)**, haematoxylin-eosin, x200), and a considerable number of foam cells **(E)**, haematoxylin-eosin, x200) and some Creutzfeldt cells **(F)**, haematoxylin-eosin, x200) are observed in the acute phase; in the chronic phase, inflammatory cells gradually migrate to the edge of the lesion **(G)**, luxol-fast blue, x400). In NMOSD and MOGAD patients, the serum immunofluorescence stain is positive for AQP4-IgG **(H)** and MOG-IgG **(I)**, respectively.

Of the 72 biopsied patients, 47 were in the other etiology group, 16 in the MS group, 5 in the MOGAD group, 2 in the Balo group, 1 in the ADEM group, and 1 in the NMOSD group. The histopathological examination of the patient’s brain biopsy tissue revealed that MS, NMOSD, MOGAD, and ADEM all exhibit demyelination with inflammatory cell infiltration, and some patients with sever CNS inflammation could demonstrate acute axonal injury. Specific antibodies like AQP4-IgG and MOG-IgG are necessary for the distinction of these illnesses. Among the brain tissues of the 2 Balo patients we collected, a typical laminar pattern between demyelination and myelin preservation area was seen in one patient, and the other patient had a histopathological presentation similar to that of MS (**Figure 1**).

### Imaging findings

The MRI of the brain revealed that 54 patients (46.5%) had a single lesion, while the remaining 62 patients (53.4%) had multiple lesions. And our cohort had 73 patients with infiltrative lesions, 28 ring-shaped, 4 megacystic, and 11 Balo-like lesions. Localization: cortex/subcortical in 88 cases, periventricular white matter in 72 cases, deep gray matter in 46 cases, and infratentorial in 41 cases. And the spinal cord MRI was available in 111/116 patients, and 15/111 patients had spinal cord lesions (cervical cord involvement in 6 cases, thoracic cord involvement in 4 cases, cervical cord and thoracic cord involvement in 4 cases, whole spinal cord involvement in 1 case). On T2WI, the median size of cerebral lesions was 4 cm (range: 2cm-11.4cm).

### Etiological classification and relapse

**Figure 2:**
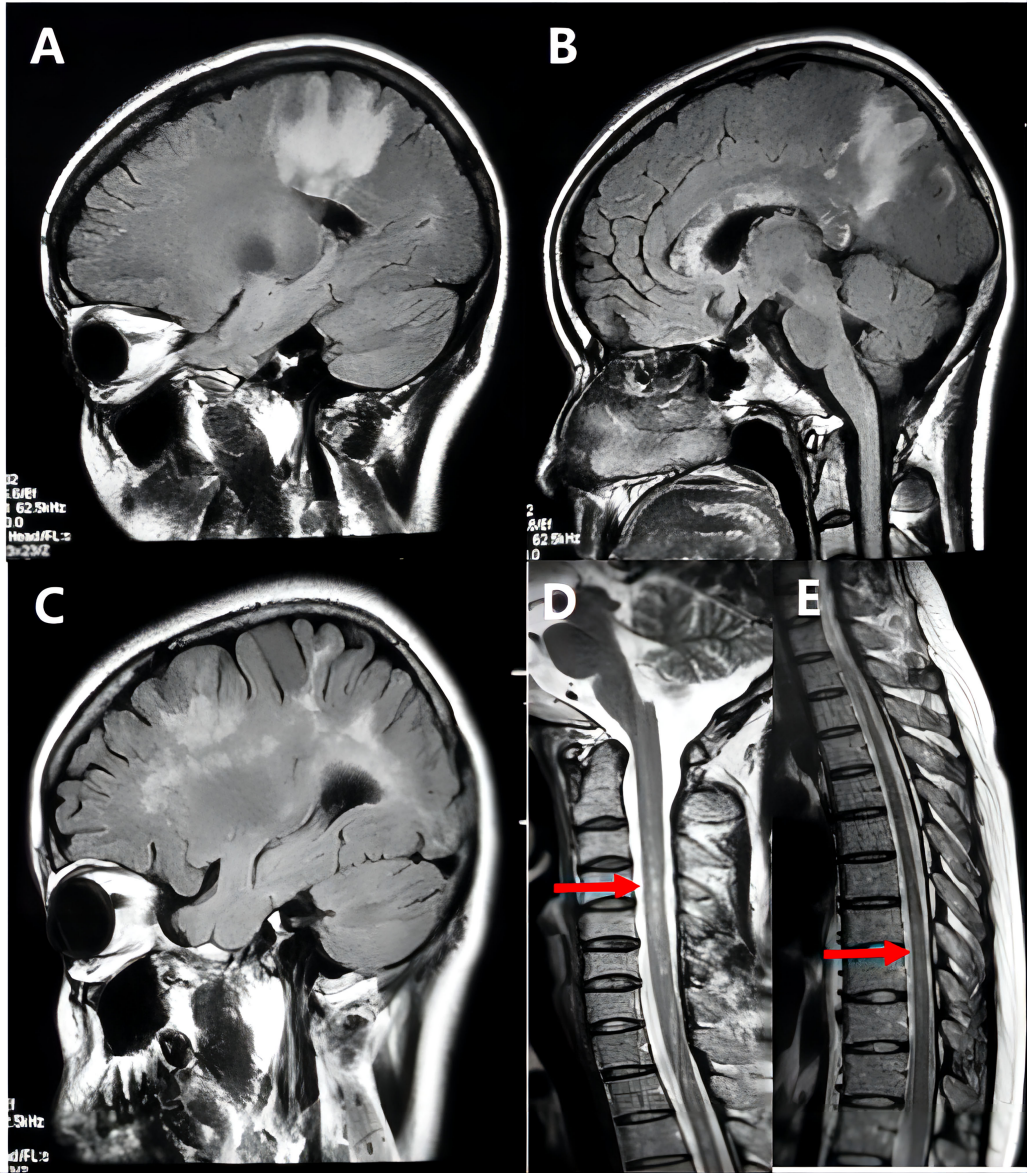
Representative example of tumefactive neuromyelitis optica spectrum disorders (NMOSD). A 26-year-old woman’s MRI scan at the disease onset showed extensive hyperintensity on T2 FLAIR images in the patient’s frontal lobe, parietal lobe, and corpus callosum **(A, B)**, and the lesions gradually decreased after treatment; after 4 months of the first attack, the patient developed more brain lesions than before **(C)**, and spinal MRI revealed long-phase abnormal signal at C3-T7 **(D, E)**. And the patient’s serum anti-AQP4 antibody was strongly positive.

**Figure 3:**
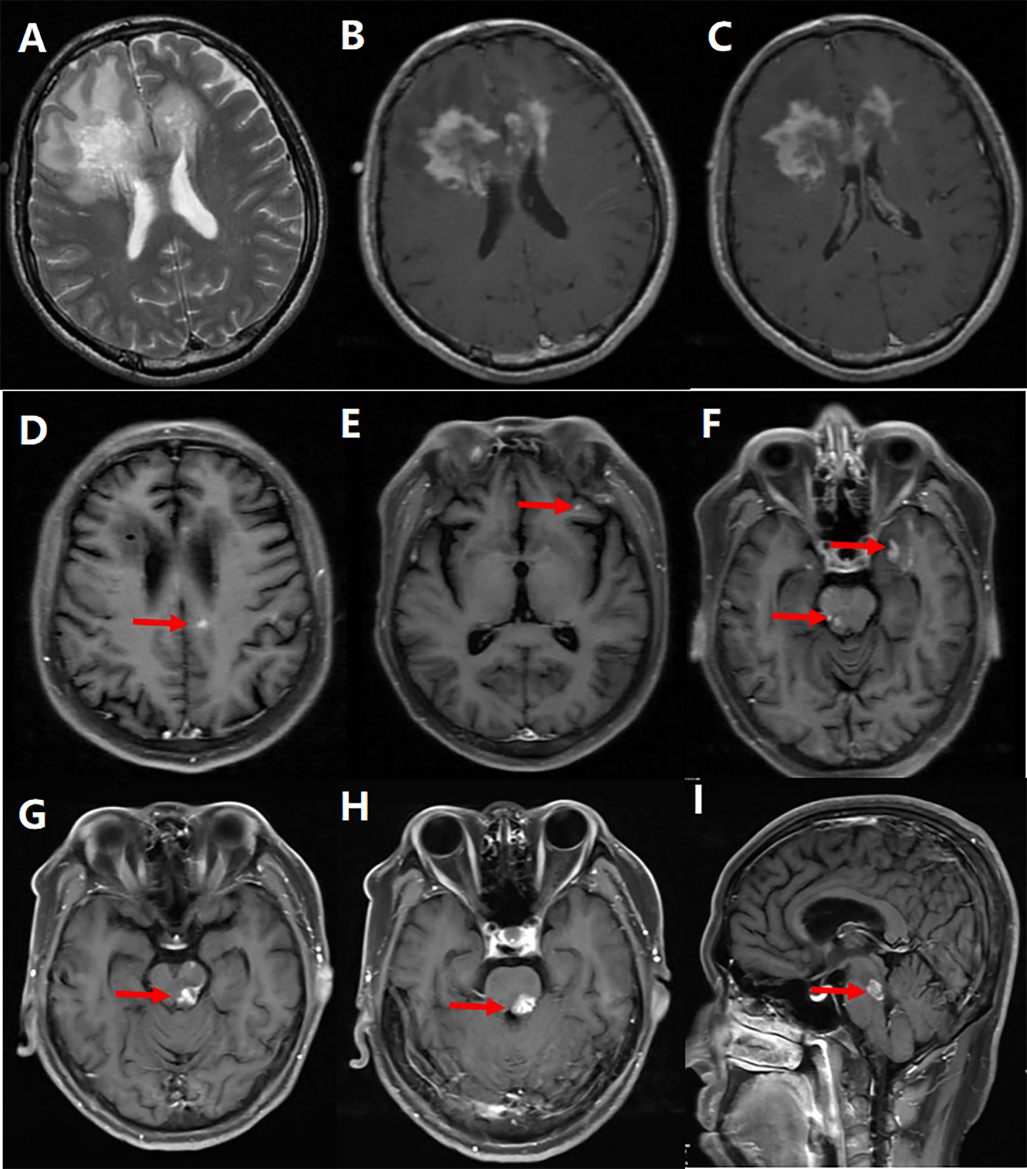
Representative example of tumefactive multiple sclerosis (MS). A 44-year-old man’s MRI scan at the first attack revealed an extensive hyperintensity on T_2_-weighted **(A)** and contrast-enhanced T_1_-weighted **(B, C)** images extending from the left frontal lobe through the corpus callosum knee to the left frontal lobe, and the lesions on the contrast enhancement images gradually disappeared after treatment; 7 months after the onset, some small patchy lesions developed in the right lateral paraventricular **(D)**, frontal **(E)**, temporal, and left midbrain **(F)**, which gradually disappeared after treatment; The second relapse occurred 19 months after the first episode, and the patient’s brainstem developed a new patchy lesions **(G–I)**.

**Figure 4:**
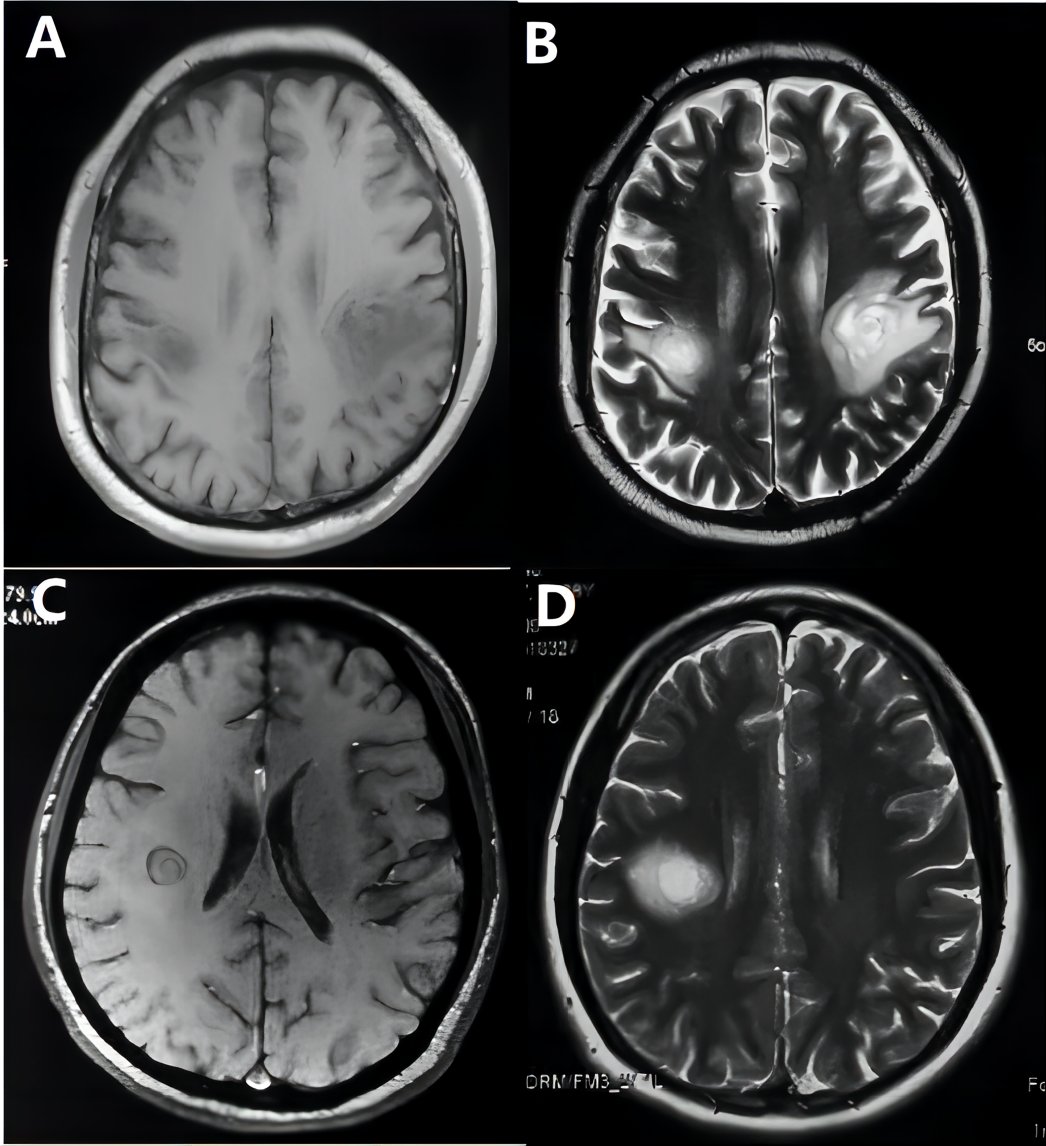
Balo images on T1-weighted **(A, C)** and T2-weighted **(B, D)** MRI of two patients.

During the 72-month (range: 6–228months) follow-up period, six patients were diagnosed with NMOSD (**Figure 2**), including one with ADEM onset and recurring AQP4-positive patients who was subsequently categorized as NMOSD. Thirty-three patients met the McDonald criteria for MS in 2017 (**Figure 3**) (12). Ten patients were diagnosed with MOGAD, six were diagnosed with Balo (**Figure 4**), and one was diagnosed with ADEM. In addition, another sixty individuals still have no clear etiology (**Table 1**).

**Table 1:** clinical date on TDLs with different etiologies.

	MS n=33	NMOSD n=6	MOGAD n=10	Balo n=6	ADEM n=1	The other etiology group (n=60)		
						Total	Isolated	Recurrent
average age at onset	37.3 ± 14.5	26.5 ± 9.2	40.8 ± 14.8	45.6 ± 11.2	5	37.4 ± 13.5	38.0 ± 13.8	34.9 ± 12.5
Gender M:F	1:0.6	1:5	1:1.5	1:5	F	1:1.14	1:1.2	1:0.86
median course of disease (month)	2(1,3)	3(2,5)	2(1.2,2)	2(1,2)	1.5	2(1,3)	1.5(1,2.3)	2(1,4)
median EDSS score	4(3, 5.9)	6(2.8, 8.3)	4.5(2.5, 6.7)	6(3, 7)	2	3(1.6, 6)	3(2, 6)	3(1.25, 5)
Lesion location								
cortex/subcortical	28/33	4/6	10	5/6	1	40/60	34/47	6/13
periventricular white matter	26/33	5/6	5/10	5/6	1	30/60	24/47	6/13
deep gray matter	16/33	3/6	6/10	1/6	N	20/60	19/47	1/13
infratentorial	18/33	3/6	3/10	N	N	17/60	12/47	5/13
spinal cord	8/33	2/6	2/10	1/6	1	1/60	1/42	N
Lesion size (cm)	2.4(2, 4.5)	4(2.5,4.1)	4.5(3.8,6.6)	4(2, 4)	5	4(3,4.6)	4(2.8, 4.8)	3.8(3.1, 4)
multiple lesions	29/32	5/6	5/10	6/7	1	16/60	12/47	4/13
OCB positivity	12/26	2/5	N	1/6	N	4/60	4/19	N
AQP-4 positivity	N	5/6	N	N	N	N	N	N
MOG positivity	N	N	10/10	N	N	N	N	N
recurrent	28/33	5/6	8/10	N	N	13/60	N	13/13

M:F, male: famale; F, famale; N, none; MS, multiple sclerosis; NMOSD, neuromyelitis optica spectrum disorders; MOGAD, myelin oligodendrocyte glycoprotein antibody-associated demyelination; ADEM, acute disseminated encephalomyelitis; OCB, oligoclonal band; AQP-4, anti-aquaporin 4.

We compared the clinical characteristics of MS, NMOSD, MOGAD, Balo, and other etiology groups. There was no statistically significant difference in age, course of disease, or EDSS score at initial onset across the various etiology groups (*P* > 0.05). But there were significantly more male patients in the MS group compared to other etiological categories (*P* = 0.033). In addition, we classified the lesions as cortical/subcortical, paraventricular, deep gray matter, infratentorial, and spinal cord, and compared the number of lesions. Multiple lesions were more prevalent in MS, NMOSD, and Balo groups than in the other etiology group (*P* < 0.001). And patients with MS, NMOSD, and MOGAD were more likely to have spinal cord involvement compared to other etiological groups (*P* = 0.016).

54 (46.6%) of the 116 patients suffered a second attack, with a median interval of 36 months between the first and second attacks (range: 1–108 months). MS group had the highest recurrence rate (84.8%), followed by NMOSD (83.3%) and MOGAD (80.0%). The median recurrence period was 7 months (range: 3-108 months) in the idiopathic group, where 13 patients (21.7%) experienced relapse. The recurrence of the other etiology group did not match the diagnostic criteria for 2017 McDonald’s MS and other demyelinating etiologies and was defined by recurrence solely at the initial lesion, solitary lesion, and absence of additional intracranial lesions. In comparison to the MS, MOGAD, and NMOSD groups, the other etiology group and the Balo group had lower rates of relapse, and there was a statistically significant difference between these groups (*P* < 0.001).

### Exploration of risk factors for relapse

The clinical, radiological, laboratory, and other data of patients with TDLs, as well as potential risk factors for disease recurrence, were initially investigated. The results revealed no statistically significant differences in gender, age, previous drinking and smoking, lesion location (cortex/subcortical, paraventricular, deep gray matter), size, laboratory examination, and biopsy between recurrent patients and non-recurrent patients (*P* > 0.05). But the clinical symptoms of multiple system function involvement, a higher EDSS score (EDSS ≥ 4), multiple lesions, diffuse infiltrative lesions, infratentorial involvement, and spinal cord involvement were associated with an increased risk of recurrence, according to the log-rank test (*P* < 0.05) (**Table S1**, in **Supplementary Material**).

Then the Cox proportional hazards model for multivariate analysis utilized the survival time and survival outcome as dependent variables and the statistically significant log rank test factors as variables to identify the final influencing factors. In the end, we found that diffuse-infiltrative lesions (*P* = 0.003, *HR* = 6.045, 95% *CI*: 1.860~19.652), multiple lesions (*P* = 0.001, *HR* = 3.262, 95% *CI*: 1.654~6.435), and infratentorial involvement (*P* = 0.006, *HR* = 2.289, 95% *CI*: 1.064-3.853) may be independent risk factors for TDLs recurrence (**Tables 2**, **3**).

**Table 2:** Multivariate analysis of risk factor screening for relapse of TDLs.

Influence factor	*P*-value	HR	95% CI
Lesion morphology (with ring like lesions as reference)	0.019*		
Balo-like	0.160	3.211	0.630 - 16.358
Macrocystic	0.199	4.817	0.438 - 53.046
Diffuse infiltrative	0.003*	6.155	1.870 - 20.252
Multiple functional systems involvement	0.431	1.418	0.594 -3.384
Multiple lesions	0.015*	2.504	1.196 - 5.246
EDSS score ≥ 4	0.659	1.165	0.592 - 2.294
Spinal cord involvement	0.316	1.480	0.687 - 3.187
Infratentorial involvement	0.032*	2.020	1.064 - 3.835

CI, confidence interval; HR, hazard ratio. *p < 0.05.

**Table 3:** Multivariate analysis of risk factor screening for relapse of TDLs.

Influence factor	*P*-value	HR	95% CI
Lesion morphology (with ring like lesions as reference)	0.018*		
Balo-like	0.162	3.030	0.640 - 14.344
Macrocystic	0.276	3.542	0.363 - 34.537
Diffuse infiltrative	0.003*	6.045	1.860 - 19.652
Multiple lesions	0.001*	3.262	1.654 - 6.435
Infratentorial involvement	0.006*	2.289	1.263 - 4.415

CI, confidence interval; HR, hazard ratio. *p < 0.05.

## Discussion

### Etiological classification

Researches have found that TDLs co-occurring or relapsing with demyelinating disorders such NMOSD, MOGAD, and ADEM, and have seen TDLs as early manifestations of these diseases with crossover in imaging and clinical symptoms (13–16). In this study, we performed a follow-up investigation of TDLs spanning a period of up to 21 years, and the etiology and risk factors for TDLs recurrence were thoroughly investigated in 116 patients. We found that about 48.3% of TDLs had a definite etiology, with MS being the most common, followed by MOGAD, Balo, and NMOSD, as well as ADEM, and patients who progressed to MS, NMOSD, and MOGAD were more likely to experience recurrence in the course of the disease. In contrast to previous studies, our research found a higher recurrence rate (46.6%) for TDLs. It could be connected to earlier research’ smaller sample sizes and shorter follow-up times, which underestimated disease recurrence (17). Furthermore, a portion of TDLs in our research still have no clear etiology, and their recurrence form was distinct from MS, NMOSD, MOGAD, and other demyelinating disorders. Jeong IH and his colleagues also described 11 patients of TDLs with unclear etiologies, five of which recurred during follow-up but still could not be clearly identified (8). Relapse in the other etiology group could happen in the original lesions or in a new place, generally affecting only one hemisphere and most often as a single lesion, which had also been emphasized in previous studies (17).

TDLs were formerly thought to represent a particular variety of MS, with 10 to 70 percent of TDLs eventually developing into MS as the disease progresses (18). It has been said that once TDLs relapses happen, their disease progression was identical to that of traditional relapsing-remitting MS (6). However, only 27.6% of patients in our study ended up in the MS group, which was somewhat different from prior studies (19, 20). We classified the etiology of patients with TDLs into six categories: MS, NMOSD, MOGAD, Balo, ADEM, and the other etiology, which is more precise than previous studies, and some subsets might be associated with MS, possibly resulting in a relative decrease in the number of patients in the MS group. And compared with other groups, the MS group had the highest recurrence rate. Furthermore, TDLs patients with persistently positive CSF-OCB should be aware of the possibility of developing MS (10). Of the 70 individuals with CSF-OCB acquired for this study, 19 had positive results, and 12 (63.2%) who were followed up on had a definite MS diagnosis. Notably, in the other etiology groups, CSF-OCB was negative in patients with recurrence and only a few patients without recurrence were positive for CSF-OCB. These recurrence-free patients with positive CSF-OCB tend to show isolated lesions on MRI and need to be differentiated from clinically isolated syndrome.

When TDLs were coupled with positive AQP4 or MOG antibodies, relapse was more likely to occur (21). NMOSD combined with CSF-OCB positivity was very rare and has only been reported in some cases previously (22, 23). We observed two patients who were positive for both AQP4 antibody and CSF-OCB, and one was positive for CSF-OCB and negative for AQP4 antibody at the beginning of the disease, but was retested positive for AQP4 antibody at the time of relapse 5 years later. In addition, we also observed a 27-year-old female who presented with recurrent visual loss and limb weakness, and whose cranial MRI showed intracranial cortical/subcortical and subcurtain occupancy-like lesions with intracranial and optic nerve enhancement and prolonged P100 latency in bilateral visual evoked potentials but who was negative for CSF-OCB and AQP-4 antibody, with a final diagnosis of NMOSD. Although positive CSF-OCB was an indicator supporting the diagnosis of MS (12), 33.3% of patients with TDLs overlapping NMOSD were positive for CSF-OCB in this study, implying that MS, NMOSD, and TDLs were not completely independent of one another.

MOGAD with TDLs as the onset presentation has been reported in the literature, but these studies are mostly case reports and focus on the pathology (21–25). In this study, we summarized 10 cases of MOGAD with TDLs as the first manifestation, and found that most of them had headache as the initial symptom, lesions were mostly located in the cortex/subcortex, and their intracranial lesions were the largest compared with other etiology groups. Previous studies showed that most patients with MOGAD respond well to hormonal therapy and have a good overall prognosis. First-line immunotherapy during the acute attack normally consists of intravenous corticosteroids, IVIG, and plasma exchange in isolation or combination. And patients with relapsing need to be treated with low-dose corticosteroids and/or chronic immunosuppressive (IS) or immunomodulatory (IM) treatments, such as azathioprine, rituximab, and mycophenolate mofetil (26, 27). However, even with IS/IM therapy, 80% patients(40/50) follow a multiphase course, and symptoms usually improve after treatment, with only a small number of patients experiencing progressive deterioration, according to a research by Jarius S and colleagues (26). In our group, all 10 patients with MOGAD used high-dose corticosteroids in the acute phase, and all of them were treated with IS treatment in the remission phase, including azathioprine (2-3 mg/(kg/d)) in 6 patients and mycophenolate mofetil (1-15 g/d) in 4 patients. After treatment, 8 patients had relapses, and only two patients recovered with no further relapses. However, the overall prognosis was good.

Studies have shown that multiple Balo like lesions can fuse into TDLs, and TDLs can also evolve into Balos conversely (28, 29). The imaging and pathology of balo, which was originally thought to be a variety of MS, were unusual, and under a microscope, the lesion’s demyelinating and myelin-retaining regions were organized in concentric layers (2). Balo is often presents with multiple lesions on MRI, preferring cortical and semi-oval centers; recurrence is possible in certain individuals. CSF-OCB could be positive. Currently, it is not recognized as a distinct demyelinating disease but rather as an imaging form subsequent to other demyelinating disorders, and there is no accepted diagnostic criteria (30). Balo’s pathology frequently exhibits demyelination and oligodendrocyte loss, which is similar to the immunological mechanism type III of MS, but the cortical gray matter remained unaffected (31, 32). There are foam macrophages, activated microglia, reactive astrocytes, and axon-loss in the demyelinating regions. Astrocytes have been proposed as the hallmark feature of this disease, because hyperplasia astrocytes are always distributed around the lesion and are closely related to oligodendrocytes (33). Two cases of Balo-like lesions in our study underwent brain biopsy. Haematoxylin-eosin staining under microscope showed multifocal lesions in the examined brain tissue, with a large number of lattice cells and astrocytes reactive proliferation, and lymphocyte “sleeve” around the blood vessels of the brain tissue. Luxol fast blue staining showed typical layered demyelination of the brain white matter in one patient, interspersed with myelin loss and the relative retention area of the myelin.

ADEM is an acute, multifocal, inflammatory demyelinating disease of the CNS that mostly affects children; cranial MRI often reveals bilateral occupancy-like lesions in the white or deep gray matter, with most cases occurring in a single course. A significant cohort research revealed that around 10.3% of ADEM patients could present with TDLs (34). According to research, ADEM patients who present with TDLs often suffer motor or cognitive sequelae, and children who have previously been diagnosed with ADEM may have behavioral problems, seizures, or repeated demyelinating episodes (35). The majority of ADEM cases were observed in pediatrics because to the age selection bias, and just one patient with ADEM onset with TDLs was gathered in our investigation. This 5-year-old child was taken to the hospital with partial body weakness and vision impairment, and cranial MRI revealed a large occupancy-like lesion with modest enhancement in the parietal and temporal lobes and an aberrant signal in the thoracic medullary lobe. Eventually, the patient recovered with a little dose of oral glucocorticoids. And there was no recurrence over the six-year follow-up period.

In conclusion, it was unclear if TDLs were specific imaging findings for demyelinating diseases such as early-stage MS, ADEM, NMOSD, and MOGAD, or whether they signify a different disease entity. This research showed that it was a complicated clinico-imaging condition with many possible causes. However, some of the causes were still not fully understood.

### Analysis of risk factors associated with TDLs relapse

We discovered that lesions of diffuse infiltration, multiple lesions, and infratentorial involvement seemed to be independent relapse risk factors. Previous research has shown that an older age at onset was a negative prognostic factor and was connected with disease progression and impairment, which might be explained by the physiological process whereby, with increasing age, damaged and senescent DNA accumulates in the cells, limiting the reserve of oligodendrocytes and reducing the capacity to produce myelin (36). However, no link was discovered in our research between age and the recurrence of TDLs. Lucchinetti and his team did not find any positive results affecting recurrence when they studied the recurrence risk factors in 85 patients with TDLs (18). But they noted that TDLs patients with multiple lesions were more likely to develop MS, which was consistent with our research. And numerous lesions and recurrence are more common in the MS group in our study. In addition, the presence of diffuse infiltrative lesions on MRI, which may progress slowly over the course of the disease, was a bad prognostic indicator, according to earlier research (11). Diffuse infiltrative lesions were linked to lower EDSS scores in patients, and steroid hormone efficacy was limited (24). And patients with spinal cord, infratentorial, or deep gray matter lesions were more likely to experience a recurrence (37–39). We confirmed that diffuse infiltrative lesions, spinal cord and infratentorial involvement were independent risk factors for recurrence in our study population.

In histopathological examination, we found that most patients showed demyelination and relative preservation of axons, but axonal damage was observed in a few patients with a severe inflammatory response, and such patients with axonal loss usually had more severe clinical symptoms and a worse prognosis than those without axonal damage. Therefore, together with previous studies (40, 41), we suppose that there is a correlation between the prognosis of patients and the degree of inflammation in the CNS, and that patients with a more severe inflammatory response tend to have a poorer prognosis. Based on our clinical experience, the IS/IM treatments should be started at an early stage of the disease in patients with intense CNS inflammation to reduce the stimulation and destruction of normal tissues by inflammation.

## Conclusions

In clinical practice, around 46.6% of TDLs relapsed, with the MS group showing the highest recurrence rate, and the imaging presence of diffuse infiltrative lesions, multiple lesions, and infratentorial involvement might be independent risk factors for relapse of TDLs. And clinicians should be aware of the possibility of recurrence if patients appear with such presentations. We found that 48.3% of TDLs had a clear etiology, such as MS, NMOSD, MOGAD, Balo, or ADEM. MS was the most common etiology. Nevertheless, despite extensive diagnostic work and long-term follow-up, there are still more than half of patients cannot be placed in any of the above categories. And the patients in the other etiology groups tend to have a monophasic course with a low recurrence rate. Our research thus emphasizes the need to investigate the etiology of patients with TDLs, and patients who meet the diagnostic criteria for MS, NMOSD, etc. should be treated in accordance with those diseases. However, the available data show that patients with unknown etiology have a better prognosis, but additional prospective studies on larger cohorts with longer follow-up are needed to assess the natural history and long-term prognosis of these patients.

## Data availability statement

The data that support the findings of this study are available from the corresponding author upon reasonable request.

## Ethics statement

This cohort study was approved by the Sixth Medical Center of PLA General Hospital ethical committee (HZKY-PJ-2022-26), and the consent of patients was obtained by the treating physician.

## Author contributions

JL, CS and QW designed the original research and revised the paper. XL, XM analyzed the data and wrote the manuscript. All authors contributed to the article and approved the submitted version.

## Funding

This work is supported by the PLA General Hospital Big Data Project (2019MBD-047).

## Conflict of interest

The authors declare that the research was conducted in the absence of any commercial or financial relationships that could be construed as a potential conflict of interest.

## Publisher’s note

All claims expressed in this article are solely those of the authors and do not necessarily represent those of their affiliated organizations, or those of the publisher, the editors and the reviewers. Any product that may be evaluated in this article, or claim that may be made by its manufacturer, is not guaranteed or endorsed by the publisher.
