# Supplementary material for: Central nervous system tumefactive demyelinating lesions: Risk factors of relapse and follow-up observations
Source: Front Immunol. 2022 Dec 1;13:1052678. doi: 10.3389/fimmu.2022.1052678 (PMC9752826; doi:10.3389/fimmu.2022.1052678)
Supplement: Supplementary file 1 [file Table_1.docx]

**Table 1 Univariate analysis of risk factor screening for relapse of TDLs**

| **influence factor** | **Number of cases** | **Recurrence free survival (months)** | ***X^2^*** | ***P*** |
| --- | --- | --- | --- | --- |
| Sexual |  |  |  |  |
| male | 55 | 48 | 0.463 | 0.496 |
| female | 61 | 108 |  |  |
| Age |  |  |  |  |
| < 35 | 53 | N | 2.306 | 0.129 |
| ≥ 35 | 63 | 48 |  |  |
| Smoke |  |  |  |  |
| Yes | 16 | N | 0.098 | 0.754 |
| No | 100 | 108 |  |  |
| Drink |  |  |  |  |
| Yes | 9 | 24 | 0.047 | 0.829 |
| No | 107 | 108 |  |  |
| Clinical symptoms of multiple system function involvement |  |  |  |  |
| Yes | 80 | 48 | 6.561 | 0.010* |
| No | 36 | N |  |  |
| EDSS Score |  |  |  |  |
| ≥ 4 | 30 | 35.16 | 5.084 | 0.024* |
| < 4 | 86 | N |  |  |
| Radiographic morphology |  |  |  |  |
| Balo-like | 11 | N |  |  |
| Macrocystic | 4 | N | 20.013 | <0.001** |
| Diffuse infiltrative | 73 | 22 |  |  |
| Ring-like | 28 | N |  |  |
| Number of lesions |  |  |  |  |
| Isolate | 54 | N | 14.406 | <0.001** |
| Multiple | 62 | 21 |  |  |
| Lesion location (cortical/subcortical) |  |  |  |  |
| With | 88 | 108 | 0.024 | 0.877 |
| Without | 28 | N |  |  |
| Lesion location (periventricular) |  |  |  |  |
| With | 72 | 60 | 1.357 | 0.244 |
| Without | 44 | 108 |  |  |
| Lesion location (deep gray matter) |  |  |  |  |
| With | 46 | 96 | 0.005 | 0.942 |
| Without | 70 | 108 |  |  |
| Lesion location (infratentorial) |  |  |  |  |
| With | 41 | 19 | 14.009 | <0.001** |
| Without | 75 | N |  |  |
| Spinal cord (111 patients) |  |  |  |  |
| with | 15 | 6.33 | 5.667 | 0.017* |
| without | 96 | 108 |  |  |
| Lesion sizeon T2WI (cm) |  |  |  |  |
| [2, 3) | 39 | 22 |  |  |
| [3, 4) | 11 | 35 | 4.354 | 0.226 |
| [4, 5) | 43 | N |  |  |
| ≥ 5 | 23 | N |  |  |
| CSF-Pressure (70 patients) |  |  |  |  |
| Abnormal | 23 | 108 | 0.816 | 0.366 |
| Normal | 47 | 22 |  |  |
| CSF-WBC (70 patients) |  |  |  |  |
| Abnormal | 13 | 19.6 | 0.828 | 0.363 |
| Normal | 57 | N |  |  |
| CSF-Glu (70 patients) |  |  |  |  |
| Abnormal | 9 | N | 0.653 | 0.419 |
| Normal | 61 | 60 |  |  |
| CSF-Chloride (70 patients) |  |  |  |  |
| Abnormal | 13 | 35.16 | 2.641 | 0.104 |
| Normal | 57 | 108 |  |  |
| CSF -Protein (70 patients) |  |  |  |  |
| Abnormal | 23 | N | 0.029 | 0.866 |
| Normal | 47 | 60 |  |  |
| OB (70 patients) |  |  |  |  |
| Positive | 19 | 22 | 0.077 | 0.781 |
| Negative | 51 | 35.16 |  |  |
| Biopsy |  |  |  |  |
| Yes | 72 | N | 3.332 | 0.068 |
| No | 44 | 48 |  |  |

CSF, cerebrospinal fluid; WBC, white blood cell; N, none. **p* < 0.05; ***p* < 0.001.
